# A deep learning based approach for prediction of *Chlamydomonas reinhardtii* phosphorylation sites

**DOI:** 10.1038/s41598-021-91840-w

**Published:** 2021-06-15

**Authors:** Niraj Thapa, Meenal Chaudhari, Anthony A. Iannetta, Clarence White, Kaushik Roy, Robert H. Newman, Leslie M. Hicks, Dukka B. KC

**Affiliations:** 1 Department of Computational Data Science and Engineering, North Carolina A&T State University, Greensboro, NC USA; 2 Department of Chemistry, University of North Carolina at Chapel Hill, Chapel Hill, NC USA; 3 Department of Computer Science, North Carolina A&T State University, Greensboro, NC USA; 4 Department of Biology, North Carolina A&T State University, Greensboro, NC USA; 5 Electrical Engineering and Computer Science Department, Wichita State University, Wichita, KS USA

**Keywords:** Machine learning, Protein function predictions

## Abstract

Protein phosphorylation, which is one of the most important post-translational modifications (PTMs), is involved in regulating myriad cellular processes. Herein, we present a novel deep learning based approach for organism-specific protein phosphorylation site prediction in *Chlamydomonas reinhardtii*, a model algal phototroph. An ensemble model combining convolutional neural networks and long short-term memory (LSTM) achieves the best performance in predicting phosphorylation sites in *C. reinhardtii.* Deemed Chlamy-EnPhosSite, the measured best AUC and MCC are 0.90 and 0.64 respectively for a combined dataset of serine (S) and threonine (T) in independent testing higher than those measures for other predictors. When applied to the entire *C. reinhardtii* proteome (totaling 1,809,304 S and T sites), Chlamy-EnPhosSite yielded 499,411 phosphorylated sites with a cut-off value of 0.5 and 237,949 phosphorylated sites with a cut-off value of 0.7. These predictions were compared to an experimental dataset of phosphosites identified by liquid chromatography-tandem mass spectrometry (LC–MS/MS) in a blinded study and approximately 89.69% of 2,663 *C. reinhardtii* S and T phosphorylation sites were successfully predicted by Chlamy-EnPhosSite at a probability cut-off of 0.5 and 76.83% of sites were successfully identified at a more stringent 0.7 cut-off. Interestingly, Chlamy-EnPhosSite also successfully predicted experimentally confirmed phosphorylation sites in a protein sequence (e.g., RPS6 S245) which did not appear in the training dataset, highlighting prediction accuracy and the power of leveraging predictions to identify biologically relevant PTM sites. These results demonstrate that our method represents a robust and complementary technique for high-throughput phosphorylation site prediction in *C. reinhardtii.* It has potential to serve as a useful tool to the community. Chlamy-EnPhosSite will contribute to the understanding of how protein phosphorylation influences various biological processes in this important model microalga.

## Introduction

Phosphorylation is one of the most widely studied post-translational modifications (PTMs) and plays a major role in signaling in myriad biological pathways. Experimental approaches for the detection of protein phosphorylation include liquid chromatography-tandem mass spectrometry (LC–MS/MS)^1,2^, radioactive chemical labeling^3^, and immunological detection, such as chromatin immunoprecipitation^4^ and western blotting^5^. Among these, only LC–MS/MS has the ability for large-scale, discovery-based phosphoproteomics but requires enrichment strategies for robust phosphorylation site identification as protein phosphorylation is transient, sub-stoichiometric, and can occur on very low abundance proteins. MS-based phosphoproteomics experiments are often costly, time-consuming, and labor-intensive. Therefore, computational predictions of phosphorylation sites offer an attractive complement to experimental-based approaches.

Machine learning (ML) approaches have been developed for prediction of phosphorylation sites recently^6–8^. These methods use manually extracted features and integrate feature selection or incorporate evolutionary information. However, model performance greatly depends on the type of features provided. There is potential for biases against features that were not considered or were unknown altogether. Until all features contributing to phosphorylation are studied or generated, the true potential of these feature-based ML models remains limited.

Deep learning (DL) models have recently been used to predict various PTMs in proteins. Unlike ML-based models, DL architectures do not require manual feature extraction. For instance, MusiteDeep^9^ is a DL-based predictor that utilizes one-hot encoding and convolutional neural networks (CNN)^10^ with attention layer, and exhibited improved performance compared to previous feature-based models. Recently, DeepPhos^11^ improved upon the performance of MusiteDeep, utilizing a multi-window approach. Both MusiteDeep and DeepPhos employ binary encoding, which is static in nature. Our previous DL-based predictors for succinylation^12^, malonylation^13^, and methylation^14^ instead utilize embedding^15^ for encoding, demonstrating significantly improved model performance compared to binary encoding.

Although numerous computational tools for phosphorylation site prediction have appeared in recent years, most are not organism-specific phosphorylation predictors. Recently, there have been a few organism-specific predictors for some model organisms (NetPhosYeast^16^, PhosPhAt^17^, PhosTryp^18^, PhosphoRice^19^, Rice_Phospho1.0^20^, PreSSFP^21^). These organism-specific phosphorylation site predictors perform better than general phosphorylation site predictors.

Herein, we have focused our phosphosite prediction efforts on the unicellular alga *Chlamydomonas reinhardtii*, a model organism for studying photosynthesis, chloroplast biology, cell cycle control, and flagellar structure and function^22–26^. Its short generation time, ability to reproduce sexually or asexually, and the ease by which it can be genetically manipulated have made *C. reinhardtii* an attractive model system for genomics analysis, evolutionary studies, and biopharmaceutical applications^27^. Considerable efforts have been focused on understanding how its biological processes are influenced by protein post-translational modifications^28–32^. Among these, interest in protein phosphorylation’s role in regulating *C. reinhardtii* cellular signaling arose with early studies detecting 52 phosphorylation sites in the eyespot^33^ and 126 phosphorylation sites in the flagella^34^. More recently, the phosphoproteome was extensively characterized, identifying 15,862 unique phosphosites with numerous phosphoproteins in key biological pathways^35^. While these studies suggest that protein phosphorylation plays an important role in regulating many cellular processes in *C. reinhardtii,* significant gaps still exist in our understanding of its phosphoproteome.

In this regard, we developed Chlamy-EnPhosSite, an organism specific DL-based predictor for *C. reinhardtii,* based on an ensemble approach, combining CNN and long short-term memory (LSTM)^36^ models. The performance of our model was benchmarked using independent test sets and demonstrated superior performance for prediction of *C. reinhardtii* phosphorylation sites than feature-based and non-organism specific models. In addition, Chlamy-EnPhosSite was also applied to predict novel sites of phosphorylation within the entire *C. reinhardtii* proteome. Our predictions were compared to a dataset of phosphosites^32^ identified by LC–MS/MS in a blinded study. These studies demonstrate that Chlamy-EnPhosSite is able to effectively predict novel sites of phosphorylation.

## Materials and methods

### Dataset

Phosphorylation sites for our benchmark dataset were identified *C. reinhardtii* serine (S), threonine (T), and tyrosine (Y) phosphorylation sites obtained from Wang et al.^35^. All phosphorylation sites captured in this dataset have been experimentally detected. This dataset was cross-referenced with the Joint Genome Institute’s *Chlamydomonas reinhardtii* database v.5.6 (19,523 entries, accessed 02/2020) appended with the NCBI chloroplast and mitochondrial databases (chloroplastic-NCBI: BK000554, mitochondrial-NCBI: NC_001638.1, 77 entries, accessed 02/2020) to obtain complete protein sequences^37^.

Positive windows were generated with the provided phosphorylated sites in the middle and an equal number of amino acids upstream and downstream. Any remaining S, T, and Y sites that were not phosphorylated in the dataset were used to generate negative windows. In those cases where the phosphosite was located near the extreme N- or C-termini of the proteins, pseudo-residues '-' were added to the windows to maintain proper window size. Duplicates were removed from both the positive and negative datasets. Finally, to generate the combined ST dataset, the S and T datasets were combined. Table 1 shows the total number of positive and negative sites generated.

**Table 1:** Positive and negative windows for phosphorylation in *Chlamydomonas reinhardtii*.

Phosphorylation Sites	Positive	Negative
S	17,732	361,218
T	3,951	213,802
Y	167	73,538
ST	21,683	434,756

The positive and negative datasets for S, T, and ST were further divided using an 80:20 ratio to generate the training and independent test datasets, respectively. Further independent test was not carried out for Y due to its smaller dataset. Due to an imbalance in the datasets, both training and independent test datasets were balanced using under-sampling. Under-sampling trims the negative dataset randomly to match the number of positive datasets. This is done to prevent any biases in the model that may develop towards positive or negative sites.

### Encoding

Traditional methods generally require manual feature extraction from window sequences, which are then fed into classification algorithms, such as ML or DL models. In contrast, our DL methods take window sequences directly as an input after the encoding.

MusiteDeep^9^ utilizes one-hot encoding, which is basically binary encoding for the protein sequences. For example, Alanine (A) is represented as 100000000000000000000, Arginine (R) is represented as 010000000000000000000, and so on. However, PTM classification models such as DeepSuccinylSite^12^, DL-Malosite^13^, and DeepRMethylSite^14^ implemented an embedded encoding scheme^15^ with better performance metrics than one-hot encoding. In this study, we used an embedding layer for the encoding of protein sequences.

First, the 20 canonical amino acids and one pseudo-residue '-' were converted into specific integers ranging from 0 to 21. These are the inputs for the embedding layer that lies at the beginning of our DL architecture. Initially, the embedding layer contains random weights or values. With subsequent epochs, it learns better vector-based representations during training. Identities are preserved, with each vectorization being an orthogonal representation in another dimension. Unlike static one-hot encoding, embedding is a dynamic encoding. The key arguments in the embedding layer are output_dim (size of vector space) and input_length (size of input windows). Hence, the output from the embedding layer has dimension input_length × output_dim.

### Deep learning models

CNN^10^ and LSTM^36^ were used as base DL models in this study. Likewise, the ensemble model^38^ named Chlamy-EnPhosSite was developed by combining these base DL models to obtain better results. A multi-window CNN model named Chlamy-MwPhosSite was also developed that comprises multiple CNN models based on different window sizes.

#### Convolutional neural network (CNN)

The encoded output from the embedding layer is fed into a 2D convolutional layer with 128 filters. Filter size is selected in a way that includes the phosphorylation site in the middle in every stride. For example, window size 53 will have a filter size of 27 × 3. The activation function used is ReLU. Padding was disabled in this layer to reduce training time without a drop in performance. The dropout layer was used to minimize overfitting. Thereafter, a 2D max-pooling layer was used with size 2 × 2. The output was fed into the last convolutional layer with 256 filters. Filter size was kept at 3 × 3 with padding enabled for this layer. After one more 2D max-pooling layer and flattening, the total features extracted were 6144. These features were fed into the dense layer with three hidden layers and the final output layer. SoftMax was used as an activation function for the final output layer. The parameter information for the CNN model is given in Table 2. Model Checkpoint function was used to extract the best model out of all the epochs based on the validation dataset with the highest accuracy and lowest loss.

**Table 2:** Parameters description of CNN model with embedding layer.

Parameters	Settings
Embedding Output Dimension	21
Learning Rate	0.001
Batch Size	256
Epochs	50
Dropout	0.4
Conv2d_1 filter (filter size)	128 (27 × 3 for window size 53)
MaxPooling2d_1	2 × 2
Conv2d_2 filter (filter size)	256 (3 × 3)
MaxPooling2d_2	2 × 2
Flatten_1	Output = 6144
Dense_1	768
Dense_2	256
Dense_3	64
Output layer activation function	Softmax
Checkpointer	Best validation accuracy

As mentioned previously by Kingma et al.^39^, Adam was used as the optimizer for our architecture. Adam utilizes adaptive learning rates to measure individual learning rates for each parameter. Since this classification is a binary classification problem, binary cross-entropy, which is the measure of uncertainty associated with a given distribution, was used as the loss function. The binary cross-entropy is given by:

1 $$ - \frac{1}{N}\mathop \sum \limits_{i = 1}^{N} \left[ {y_{i} \log \left( {\hat{y}_{i} } \right) + \left( {1 - y_{i} } \right)\log \left( {1 - \hat{y}_{i} } \right)} \right] $$

where y is either 1 for positive or 0 for negative and $\hat{y}_{i }$ is the predicted probability of the site being positive for all N points.

#### Long short-term memory (LSTM)

The encoded output from the embedding layer was fed into the LSTM layer with a dropout of 0.4. The output from two consecutive LSTM layers was then fed into the dense layers with two hidden layers. ReLU was used as an activation function for LSTM layers while SoftMax was used for the final output layer. Adam was used as an optimizer, as described above. Model Checkpoint function was used to extract the best model out of all the epochs based on the validation dataset with the highest accuracy and lowest loss. The parameter information for the LSTM model is given in Table 3.

**Table 3:** Parameters description of LSTM model with embedding layer.

Parameters	Settings
Embedding Output Dimension	21
Learning Rate	0.001
Batch Size	256
Epochs	50
LSTM layer 1 memory units	128
LSTM layer 2 memory units	64
LSTM layer 2 dropout	0.4
Dense layer 1	128
Dropout	0.4
Dense layer 2	64
Dropout	0.4
Output layer activation function	Softmax
Checkpointer	Best validation accuracy

#### Multi-windows CNN model

**Figure 1:**
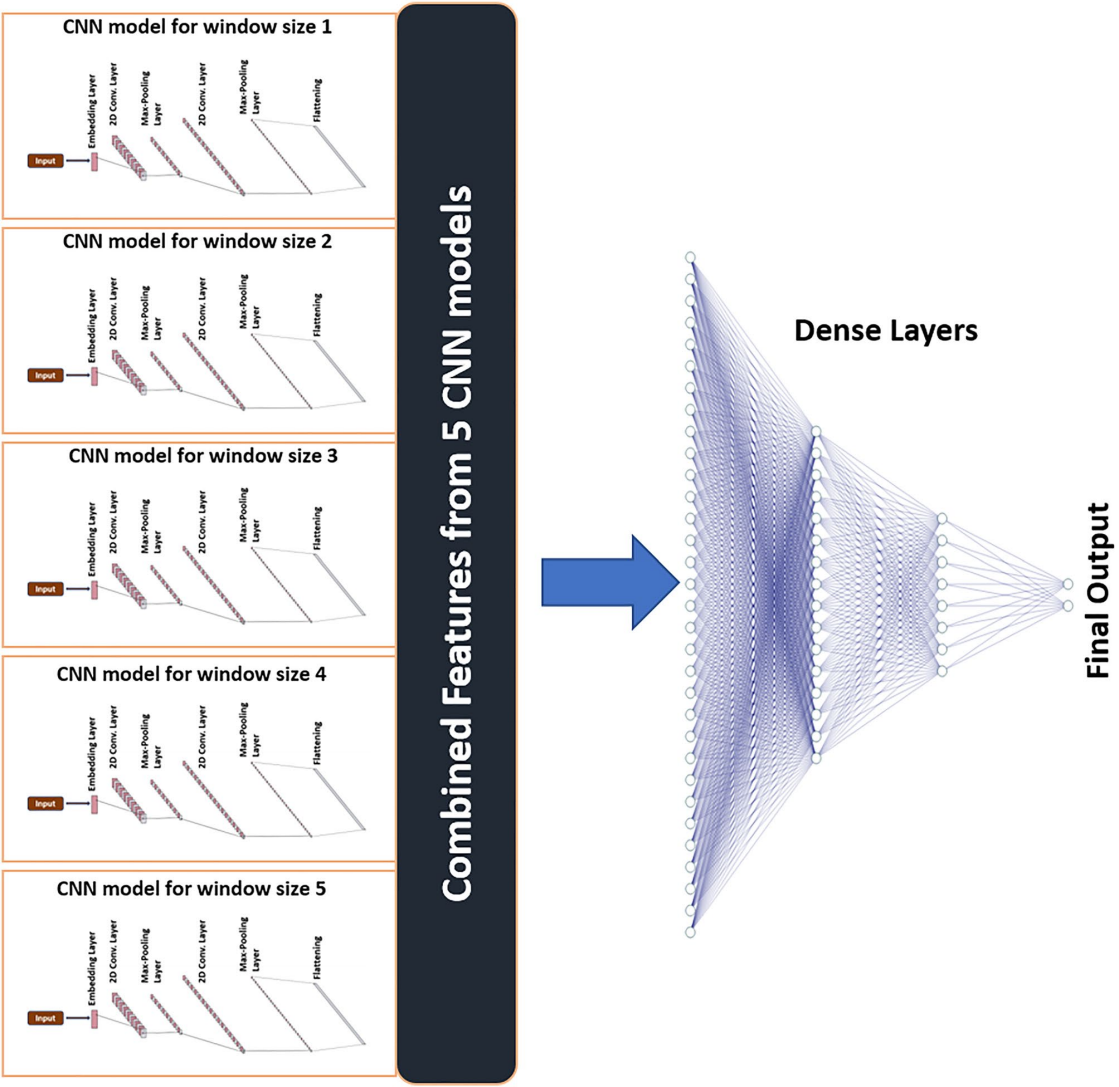
Multi-windows model Chlamy-MwPhosSite combining features from five models with different window sizes.

The Multi-windows CNN model, which we have named Chlamy-MwPhosSite, merges features extracted by our CNN models for different window sizes. It then feeds the combined features into the dense layer, and provides the output. It categorically ends the need to choose one window size for the classification, thus strengthening the model. As shown in Fig. 1, Chlamy-MwPhosSite combines features from five different CNN models with different window sizes.

#### Ensemble model

**Figure 2:**
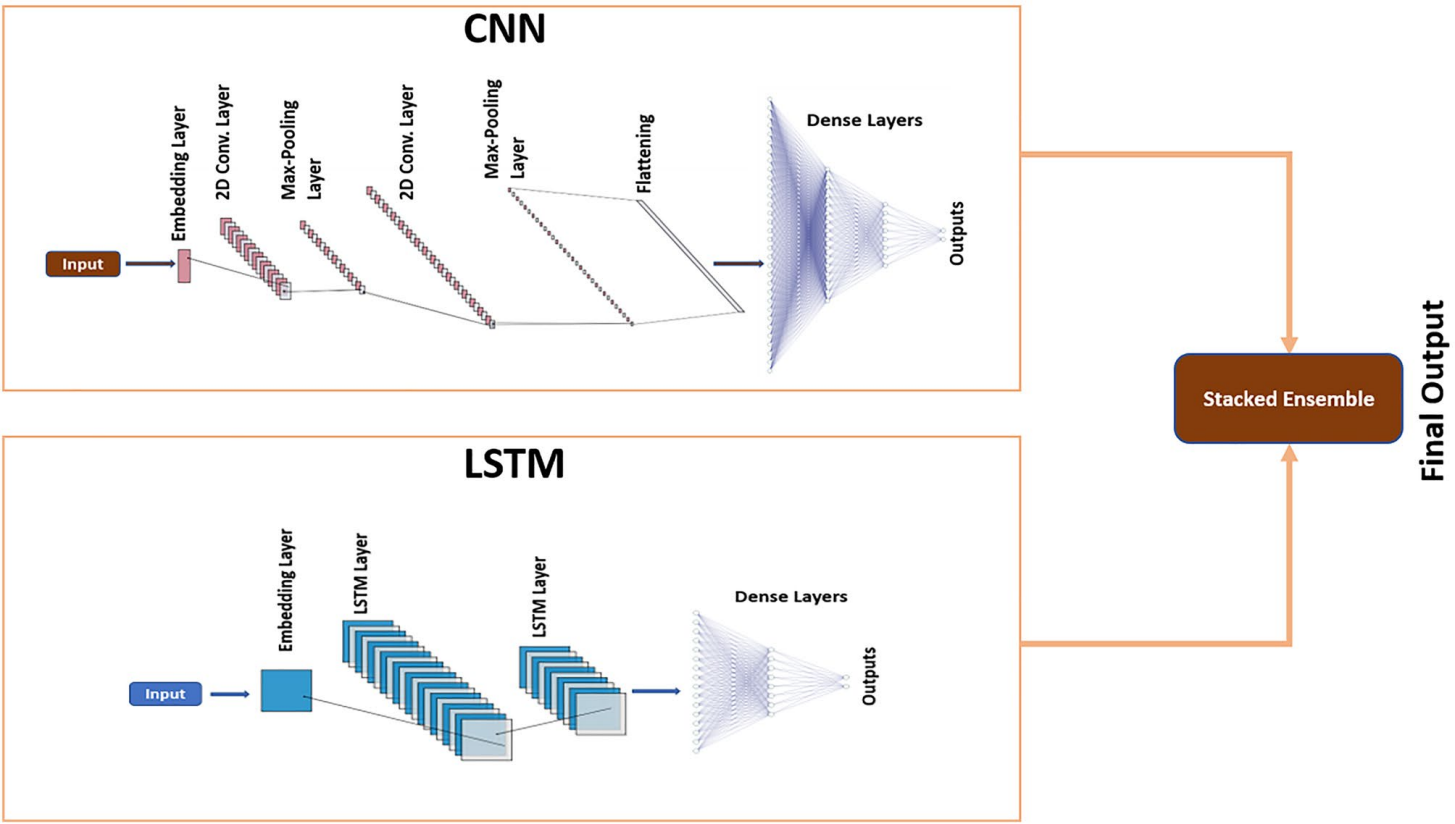
Ensemble model Chlamy-EnPhosSite combining CNN and LSTM models with stacking.

In this study, the Ensemble model, which we have named Chlamy-EnPhosSite, merges CNN and LSTM models using stacking^38^, as shown in Fig. 2. The stacked ensemble uses a meta-learning algorithm to find the best combination of these models. The meta models are trained on the results obtained from CNN and LSTM models. In our case, we used neural networks to combine them.

### Model evaluation and performance metrics

In this study, tenfold cross-validation was used to evaluate the performance of the model and to determine its robustness and generalizability. During tenfold cross-validation, the data are partitioned into ten equal parts. Then, one-part is left out for validation while training is performed on the remaining nine parts. This process is repeated until all parts are used for validation. For the results of tenfold cross-validation, unless otherwise noted, all performance metrics are reported as the mean value ± standard deviation.

Confusion Matrix, Matthew's Correlation Coefficient (MCC), and Receiver Operating Characteristics (ROC) curve were used as performance metrics. The ROC curve is a graphical plot that illustrates the diagnostic ability of a binary classifier, whereas area under the curve (AUC) represents the degree or measure of separability. Since this is a binary classification problem, the confusion matrix size is 2 × 2 composed of true positives (TP), true negatives (TN), false positives (FP), and false negatives (FN). Its diagonal elements are true predicted values. Other metrics calculated using these variables were accuracy, sensitivity (i.e., the true positive rate), and specificity (i.e., the true negative rate).

2 $$ Accuracy = \frac{TP + TN}{{TP + TN + FP + FN}} \times 100 $$

3 $$ Sensitivity = \frac{TP}{{TP + FN}} \times 100 $$

4 $$ Specificity = \frac{TN}{{TN + FP}} \times 100 $$

5 $$ MCC = \frac{{\left( {TP} \right)\left( {TN} \right) - \left( {FP} \right)\left( {FN} \right)}}{{\sqrt {\left( {TP + FP} \right)\left( {TP + FN} \right)\left( {TN + FP} \right)\left( {TN + FN} \right)} }} $$

## Results

### Performance of models trained on non-organism specific phosphorylation sites

To compare our models with existing phosphorylation site prediction models, we compared the performance of Chlamy-EnPhosSite and Chlamy-MwPhosSite to that of existing DL-based predictors. For these studies, we used the combined ST dataset used during the development of MusiteDeep^9^ as a benchmarking dataset. This was done due to DeepPhos's better overall performance with respect to MCC and other performance metrics compared to MusiteDeep. All the models were trained on the same training dataset and tested on the same independent test dataset. Interestingly, for all performance metrics tested, our base models exhibited higher values than DeepPhos. This may suggest that embedding provides additional enhancements in model performance compared to the binary encoding strategy used by previous DL-based phosphosite predictors. Further performance gains were observed for Ensemble (CNN Multi-window) and Ensemble-stacking (CNN + LSTM) for most metrics (Table 4). Rice_Phospho 1.0^20^, an organism-specific model, has obtained significantly better MCC (0.62). Hence, for further possible improvement in performance for *C. reinhardtii,* we approach towards analysis of organism specific phosphorylation sites prediction.

**Table 4:** Performance metrics of different models using an independent test dataset for general phosphorylation sites S and T.

Models	Sensitivity	Specificity	Accuracy	AUC	MCC
DeepPhos	0.72	0.73	0.75	0.82	0.52
LSTM with embedding	0.80	0.76	0.78	0.78	0.56
CNN with embedding (Trained on MusiteDeep Dataset)	0.83	0.75	0.79	0.87	0.58
Ensemble (CNN Multi-window)	0.84	0.76	0.80	0.88	0.60
Ensemble-stacking (CNN + LSTM)	0.86	0.73	0.79	0.88	0.59

### Model evaluation using manually extracted features

Next, we also investigated the performance of a ML model (Random Forest) and DL model on manually extracted features from the *C. reinhardtii* dataset. We generated physicochemical-based features like Pseudo Amino Acid Composition (PAAC), k-Spaced Amino Acid Pairs (AAP) and Composition, Transition and Distribution (CTD) as well as autocorrelation features like Moreau-Broto Autocorrelation (MBA) and Entropy Features, such as Shannon Entropy (SE), Relative Entropy (RE), and Information Gain (IG). Using Random Forest (RF), we selected 178 optimized features. Using these features both RF and DL models were evaluated. The results are shown in Table 5. Our performance suffered using the manually extracted features, even when compared to non-organism specifics models.

**Table 5:** Performance metrics of different models applying manually extracted features using an independent test dataset for S and T.

Models	Sensitivity	Specificity	Accuracy	AUC	MCC
Random Forest (RF)	0.84	0.61	0.72	0.80	0.46
CNN	0.88	0.58	0.73	0.81	0.48

### Model development and tenfold cross-validation

We performed tenfold cross-validation using a base CNN with embedding model on different window sizes ranging from 9 to 61 on S, T and ST (Table S1-S3). Further window sizes were not analyzed due to the sheer size of the windows and the corresponding increase in the number of pseudo-residues ‘-’ that was required at higher window sizes.

**Figure 3:**
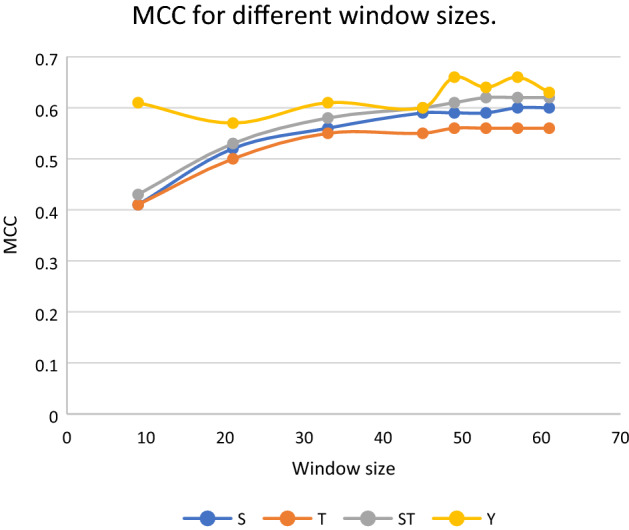
Tenfold cross-validation mean MCC of S, T, ST and Y for different window sizes.

From Fig. 3, a general trend for MCC of S, T and ST shows improvement with increasing window sizes up to around 45. Thereafter, it reaches a plateau with not much significant improvements in performance. Optimal window sizes of 57, 53 and 53 were chosen for S, T and ST respectively, for further study.

For Y, the results of tenfold cross-validation are shown in Table S4. The relatively high standard deviations observed for this dataset suggest that there is more variability in performance, which is not surprising given the smaller size of the Y dataset compared to the other datasets. From Fig. 3, MCC for Y does not follow specific pattern. For these reasons, the Chlamy-EnPhosSite and Chlamy-MwPhosSite models were not applied to the Y phosphorylation dataset, and an independent test was not performed.

### Assessment of Chlamy-EnPhosSite using independent testing

Next, an independent test was carried out with different models for S, T, and ST using 20% of each dataset that had been set aside for independent testing. For these studies, the window sizes that exhibited the best performance when evaluated by tenfold cross-validation were used respectively, as described above. For independent testing, LSTM, CNN, Chlamy-MwPhosSite, and Chlamy-EnPhosSite models were trained on the 80% of the dataset set aside for training.

**Figure 4:**
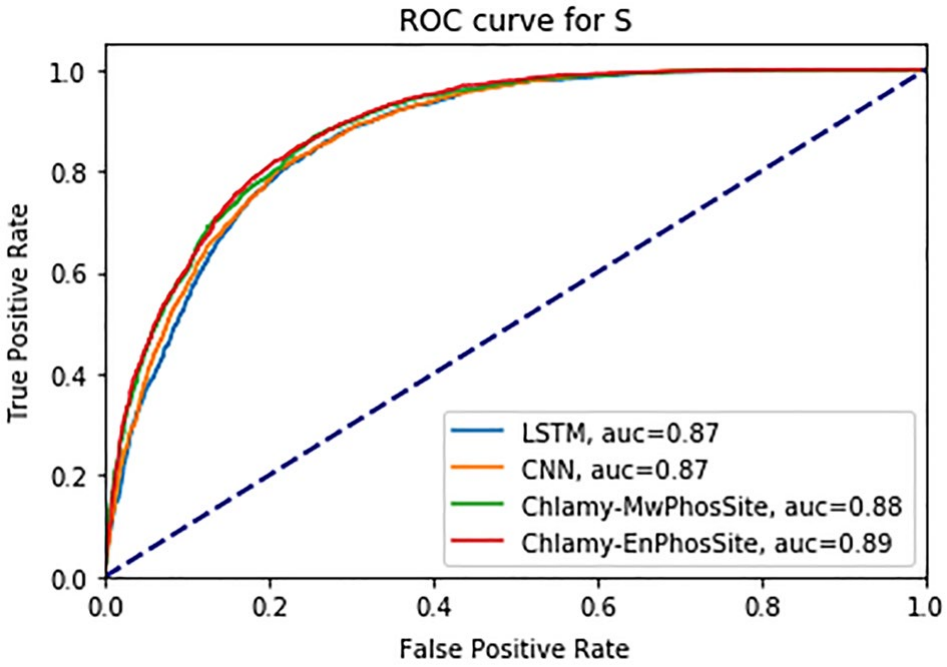
ROC curve for different DL models for S.

The results of the independent test with the S dataset are shown in Table 6 and the ROC curve is shown in Fig. 4. Both Chlamy-MwPhosSite and Chlamy-EnPhosSite exhibited improved performance compared to base models of LSTM and CNN. For instance, the highest AUC and MCC 0.89 and 0.62 respectively, were observed for Chlamy-EnPhosSite, although these values were only marginally better than those observed for Chlamy-MwPhosSite.

**Table 6:** Performance metrics of different models using an independent test dataset for S.

Models	SN	SP	ACC	AUC	MCC
LSTM with embedding	0.87	0.72	0.79	0.87	0.59
CNN with embedding	0.89	0.69	0.79	0.87	0.60
Chlamy-MwPhosSite	0.89	0.71	0.80	0.88	0.61
Chlamy-EnPhosSite	0.89	0.72	0.80	0.89	0.62

**Figure 5:**
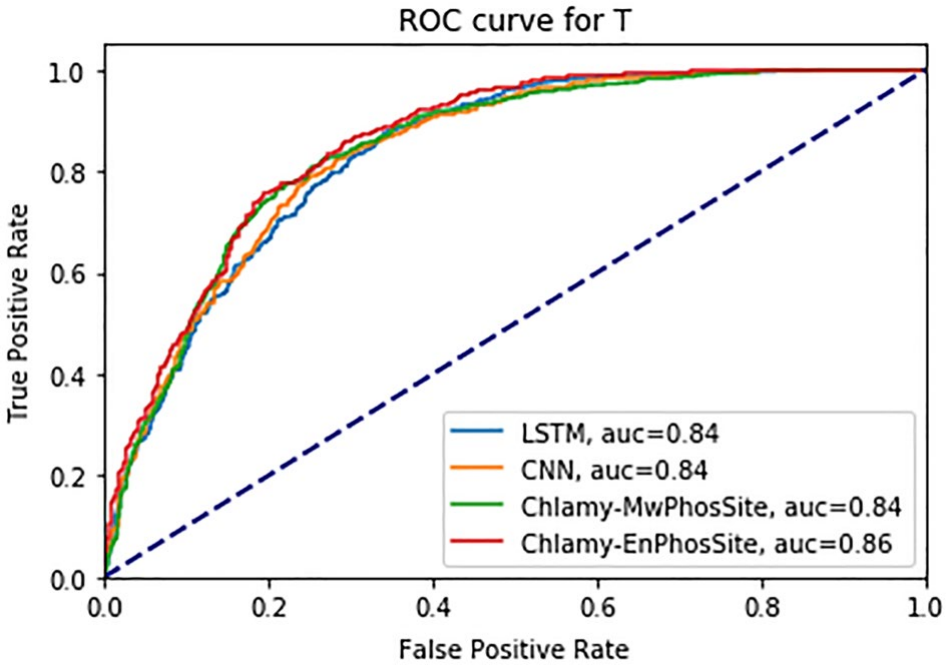
ROC curve for different DL models for T.

For the T dataset, the results of the independent test are shown in Table 7, and the ROC curve is shown in Fig. 5. Both Chlamy-EnPhosSite and Chlamy-MwPhosSite have improved performance on base models, LSTM and CNN. The best values for AUC, MCC, and SN (0.86, 0.56, and 0.92 respectively) were attained by Chlamy-EnPhosSite, whereas the best values for SP and ACC (0.79 and 0.78 respectively) were attained by Chlamy-MwPhosSite.

**Table 7:** Performance metrics of different models using an independent test dataset for T.

Models	SN	SP	ACC	AUC	MCC
LSTM with embedding	0.83	0.69	0.76	0.84	0.53
CNN with embedding	0.86	0.66	0.76	0.84	0.53
Chlamy-MwPhosSite	0.76	0.79	0.78	0.84	0.55
Chlamy-EnPhosSite	0.92	0.61	0.77	0.86	0.56

**Figure 6:**
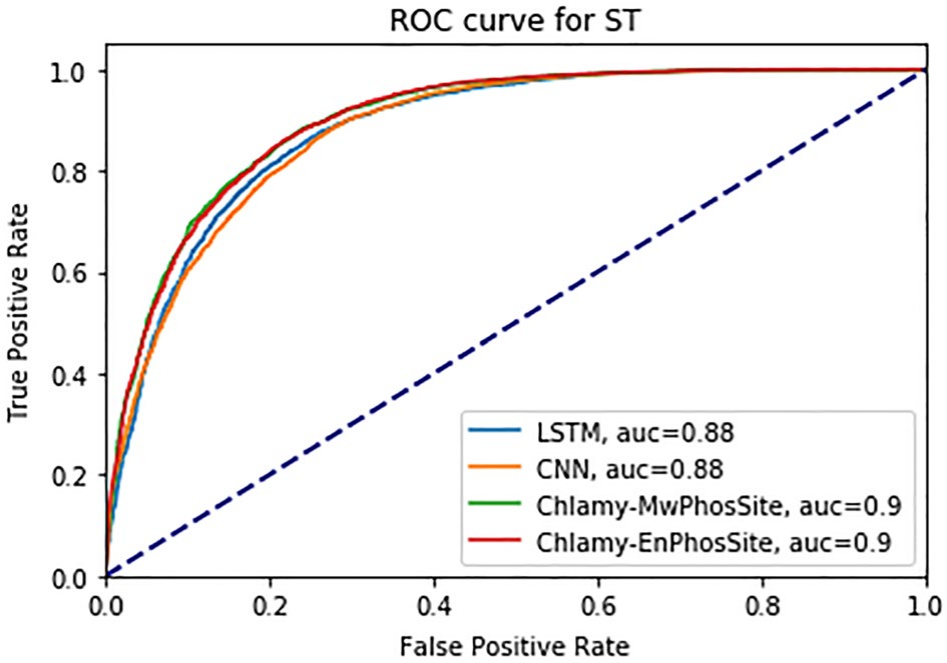
ROC curve for different DL models for S and T combined.

For the ST dataset, both Chlamy-EnPhosSite and Chlamy-MwPhosSite exhibited improved performance compared to the LSTM and CNN base models (Table 8 and Fig. 6). For model benchmarking, we also trained and tested the *C. reinhardtii* dataset using DeepPhos. It exhibited similar performance to our base CNN and LSTM models but did not perform as well as our ensemble approaches. For instance, both Chlamy-MwPhosSite and Chlamy-EnPhosSite attained AUC, MCC, and ACC of 0.90, 0.64 and 0.82, respectively. Interestingly, CNN with embedding achieved the best SN (0.91), whereas Chlamy-MwPhosSite performed the best with respect to SP (0.78).

**Table 8:** Performance metrics of different models using an independent test dataset for S and T.

Models	SN	SP	ACC	AUC	MCC
DeepPhos	0.83	0.77	0.81	0.88	0.61
LSTM with embedding	0.87	0.74	0.81	0.88	0.61
CNN with embedding	0.91	0.69	0.81	0.88	0.61
Chlamy-MwPhosSite	0.86	0.78	0.82	0.90	0.64
Chlamy-EnPhosSite	0.90	0.73	0.82	0.90	0.64

### Predicting phosphorylation sites in entire *C. reinhardtii* proteome using Chlamy-EnPhosSite

Our independent test results suggest that Chlamy-EnPhosSite (ensemble-based approach) is the best predictor (although marginally), thus we used Chlamy-EnPhosSite for subsequent analysis. To explore the utility of Chlamy-EnPhosSite for predicting novel phosphosites, we applied Chlamy-EnPhosSite to predict S and T phosphorylation sites in the full *C. reinhardtii* proteome*.* Chlamy-EnPhosSite was applied to predict phosphorylation sites on a total of 1,809,304 S/T sites and it was able to perform these predictions in about an hour using a GeForce RTX 2080 machine. With 0.5 as a probability cut-off, Chlamy-EnPhosSite predicted 499,411 phosphorylated sites and with cut-off value of 0.7, Chlamy-EnPhosSite predicted 237,949 phosphorylated sites.

In addition, we also validated the predictions made by Chlamy-EnPhosSite on the entire *C. reinhardtii* proteome using a newly generated dataset of phosphosites from *C. reinhardtii*^32^. Like the independent test sets, our model was blind to this new dataset during training, therefore these studies serve as a second, completely independent test set of S/T residues. Within this new dataset, 2,663 novel *C. reinhardtii* S and T phosphorylation sites were included since they were not present in the previous dataset. Using 0.5 as a cut-off, Chlamy-EnPhosSite was able to predict 2,362 out of 2,663 (89.69%) phosphorylated sites correctly. Using a more stringent cut-off of 0.7, Chlamy-EnPhosSite still correctly predicted 2,046 out of 2,663 (76.83%) phosphorylated sites. By further increasing the cut-off, the probability of avoiding false positives increases, but there is a trade-off with a decrease in the number of true positives. Together, these data suggest that our DL-based model, Chlamy-EnPhosSite, could be used to predict novel phosphosites in *C. reinhardtii*.

These phosphorylation site predictions can elucidate protein modulation in important signaling cascades such as the target of rapamycin (TOR) signaling pathway. The TOR kinase is a conserved master regulator of cell growth whose activity is modulated in response to nutrients, energy, and stress^40–42^. This includes regulation of protein synthesis and degradation through the control of translation, ribosome biosynthesis, and autophagy^43^. In *Arabidopsis thaliana*, TOR directly phosphorylates ribosomal protein S6 kinase (S6K), which in turn phosphorylates ribosomal protein S6 (RPS6)^44^. A method to monitor TOR activity in *C. reinhardtii* through S6K phosphorylation has been difficult to obtain because S6K phosphopeptide identification has eluded MS detection and commercial anti-phosphoS6K antibodies have failed^32,45^. Instead, antibodies against the downstream *C. reinhardtii* RPS6 phosphosite S245, a conserved site that is phosphorylated by S6K in a TOR-dependent manner in yeast and humans^46,47^, has been used as a proxy to monitor TOR activity. Validation confirmed that this site is phosphorylated in a TOR-dependent manner and can be used to monitor TOR function in *C. reinhardtii*. Interestingly, typical quantitative LC–MS/MS-based phosphoproteomics methods using TiO_2_ enrichment failed to detect RPS6-S245 phosphorylation, and this site was only detected by orthogonal enrichment strategies and extensive fractionation. However, the model described herein, Chlamy-EnPhosSite was able to predict phosphorylation on RPS6 S245 with a probability of 0.65, displaying prediction accuracy and the ease of phosphorylation site identification compared to MS-based methods. This may be extended to other kinase/signaling pathway intermediates whereby sites predicted could then lead to viable routes for validation/activity readouts in subsequent biologically focused experiments.

## Conclusion and discussions

*C. reinhardtii* is the most intensively studied and well-developed model for the investigation of a wide range of microalgal processes. These efforts have identified that phosphorylation-based regulation of proteins in *C. reinhardtii* is essential for its underlying biology. However, the characterization of this organism's phosphoproteome has been limited. Here, we have built a DL-based predictor, Chlamy-EnPhosSite, that is able to identify phosphorylation sites in *C. reinhardtii* using only the primary amino acid sequence as input. Because the DL architecture eliminates the need for manual feature extraction, these methods are less computationally expensive and are not biased toward a particular feature or set of features. Importantly, consistent with our previous studies, embedding was found to be superior to binary encoding as an encoder for protein sequences, even in our base CNN and LSTM models.

Chlamy-EnPhosSite combines CNN and LSTM models using a stacking ensemble algorithm, whereas our other approach, Chlamy-MwPhosSite (which produces similar results as Chlamy-EnPhosSite) combines features from five models (from five different windows) and feeds these data into the neural network. One of the main advantages of Chlamy-MwPhosSite is its ability to use multiple windows instead of using just one window sequence. Tenfold cross-validation was used to determine optimal window sizes between 9 to 61. Model benchmarking was performed to determine how our DL-based models compared to the state-of-the-art models. Each of our models achieved some improvement in comparison to the existing DL-based models. In fact, even our base CNN and LSTM models exhibited improvements in most metrics, which is likely a function of our embedding strategy versus the binary encoding strategies used during the development of previous models. Likewise, all four methods were systematically validated with an independent test for S, T, and ST sites. Chlamy-EnPhosSite and Chlamy-MwPhosSite both improved on the performances of CNN and LSTM models, with marginal differences in their performances compared to one another.

There are still challenges for the development of better predictors. One of the main challenges is the size of the dataset, which we saw clearly with higher variance on predictor performance for the Y dataset due to its small size. In the future, with the increase in the number of experimentally verified Y sites, model prediction performance is also likely to increase. The other challenge is the predictability of features extracted by DL models. At this point, our models have a “black-box” nature, where protein sequences are entered, and predictions are produced. However, it is imperative to know about the features learned by these models for experimental improvements. To this end, explainable DL strategies^48^ could hold the key in the future.

Chlamy-EnPhosSite and Chlamy-MwPhosSite show a substantial improvement in predictive quality over models based on manually extracted features and non-organism specific phosphorylation site predictors for *C. reinhardtii*. The performance improvement for phosphorylation site prediction in *C. reinhardtii* using Chlamy-EnPhosSite proves that models trained on organism-specific phosphorylation sites are better in predicting phosphosites for that particular organism which is in line with other organism-specific phosphorylation site predictors and highlights the importance of developing organism-specific predictors as the data for phosphorylation sites of these organisms become available. The predictions from our models may be used to guide experiments and facilitate hypothesis-driven interrogation of phosphorylation sites. Importantly, the use of these models could significantly cut down on the time and cost of phosphosite identification.

## Supplementary Information

Supplementary Information.

## Data Availability

The test model and data has been made available at http://github.com/dukkakc/Chlamy-EnPhosSite.
