# Supplementary material for: A deep learning based approach for prediction of Chlamydomonas reinhardtii phosphorylation sites
Source: Sci Rep. 2021 Jun 15;11:12550. doi: 10.1038/s41598-021-91840-w (PMC8206365; doi:10.1038/s41598-021-91840-w)
Supplement: Supplementary file 1 — Supplementary Information. [file 41598_2021_91840_MOESM1_ESM.docx]

**Supplementary Materials:**

# **A deep learning based approach for prediction of *Chlamydomonas reinhardtii* phosphorylation sites**

**10-fold cross validation for different windows:**

We performed 10-fold cross-validation using a base CNN with embedding model on different window sizes ranging from 9 to 61 on the entire dataset. For example, a window size of 61 would have either a S, T, or Y residue in the middle flanked by 30 amino acids on either side. Further windows were not analyzed due to the sheer size of the windows and the corresponding increase in the number of pseudo-residues '-' that was required at higher window sizes.

When analyzing the S dataset, the performance of the CNN model increased with increasing window sizes for nearly all performance metrics tested (i.e., sensitivity (SN), ACC, AUC, and MCC) (**Table S1**). The only exception was specificity (SP), which displayed a maximum value at lower window size of 9 as well. The mean values of MCC, AUC, and ACC at this window size were 0.60 ± 0.01, 0.88 ± 0.01, and 0.80 ± 0.01, respectively. Though the highest SN and SP values were also obtained using window size 61 and 9, respectively, the performance using window size 57 was comparable with respect to these metrics, while exhibiting the highest values of MCC, ACC, and AUC. Therefore, window size 57 for S phosphorylation sites was used for further independent testing.

Table S1. 10-fold cross-validation results for different windows for S sites prediction.

| **Window Size** | **SN** | **SP** | **ACC** | **AUC** | **MCC** |
| --- | --- | --- | --- | --- | --- |
| 61 | 0.90 ± 0.01 | 0.68 ± 0.01 | 0.79 ± 0.01 | 0.88 ± 0.01 | 0.60 ± 0.02 |
| 57 | 0.88 ± 0.03 | 0.71 ± 0.03 | 0.80 ± 0.01 | 0.88 ± 0.01 | 0.60 ± 0.01 |
| 53 | 0.88 ± 0.03 | 0.70 ± 0.04 | 0.79 ± 0.01 | 0.87 ± 0.01 | 0.59 ± 0.01 |
| 49 | 0.87 ± 0.03 | 0.71 ± 0.03 | 0.79 ± 0.01 | 0.87 ± 0.01 | 0.59 ± 0.01 |
| 45 | 0.88 ± 0.01 | 0.70 ± 0.02 | 0.79 ± 0.01 | 0.87 ± 0.01 | 0.59 ± 0.01 |
| 33 | 0.85 ± 0.03 | 0.70 ± 0.03 | 0.78 ± 0.01 | 0.86 ± 0.01 | 0.56 ± 0.01 |
| 21 | 0.82 ± 0.02 | 0.69 ± 0.03 | 0.76 ± 0.01 | 0.83 ± 0.01 | 0.52 ± 0.01 |
| 9 | 0.69 ± 0.02 | 0.71 ± 0.03 | 0.70 ± 0.01 | 0.77 ± 0.01 | 0.41 ± 0.02 |

Similar to what was observed for S, most performance metrics for the CNN model increased with increasing window size when evaluating the T dataset (**Table S2**). Beginning at window size 57, the model performance began to decline slightly for most metrics. The only exceptions was SP, which exhibited the highest value at a window size of 33. Due to its consistently high performance for most metrics, we chose window size 53 for further independent testing of T phosphorylation sites. At this window size, the mean MCC, AUC, and ACC values were 0.56 ± 0.02, 0.85 ± 0.01, and 0.78 ± 0.01, respectively (**Table S2**).

Table S2. 10-fold cross-validation results for different windows for T sites prediction.

| **Window Size** | **SN** | **SP** | **ACC** | **AUC** | **MCC** |
| --- | --- | --- | --- | --- | --- |
| 61 | 0.87 ± 0.03 | 0.67 ± 0.03 | 0.77 ± 0.01 | 0.84 ± 0.01 | 0.56 ± 0.02 |
| 57 | 0.87 ± 0.03 | 0.68 ± 0.04 | 0.77 ± 0.01 | 0.85 ± 0.01 | 0.56 ± 0.02 |
| 53 | 0.87 ± 0.02 | 0.68 ± 0.04 | 0.78 ± 0.01 | 0.85 ± 0.01 | 0.56 ± 0.02 |
| 49 | 0.86 ± 0.03 | 0.69 ± 0.04 | 0.77 ± 0.01 | 0.84 ± 0.01 | 0.56 ± 0.22 |
| 45 | 0.86 ± 0.03 | 0.68 ± 0.02 | 0.77 ± 0.01 | 0.84 ± 0.01 | 0.55 ± 0.03 |
| 33 | 0.83 ± 0.02 | 0.71 ± 0.02 | 0.77 ± 0.01 | 0.84 ± 0.01 | 0.55 ± 0.01 |
| 21 | 0.82 ± 0.05 | 0.68 ± 0.05 | 0.75 ± 0.02 | 0.81 ± 0.02 | 0.50 ± 0.03 |
| 9 | 0.72 ± 0.04 | 0.69 ± 0.04 | 0.70 ± 0.01 | 0.76 ± 0.02 | 0.41 ± 0.03 |

For the combined ST dataset, the results of 10-fold cross-validation mirrored those observed for T alone (**Table S3**). For instance, the performance of the CNN model increased as window size increased with a peak at window size 53 that started to decline thereafter. At window size 53, mean MCC, AUC, and ACC values were 0.62 ± 0.01, 0.89 ± 0.01, and 0.81 ± 0.01, respectively (**Table S3**). The highest SN was obtained using window size of 57. Due to better performance in most metrics, window size 53 was again selected for further independent testing of combined ST phosphorylation sites.

Table S3. 10-fold cross-validation results for different windows for S and T sites prediction.

| **Window Size** | **SN** | **SP** | **ACC** | **AUC** | **MCC** |
| --- | --- | --- | --- | --- | --- |
| 61 | 0.88 ± 0.01 | 0.73 ± 0.02 | 0.81 ± 0.01 | 0.88 ± 0.01 | 0.62 ± 0.01 |
| 57 | 0.90 ± 0.01 | 0.71 ± 0.02 | 0.80 ± 0.01 | 0.89 ± 0.01 | 0.62 ± 0.02 |
| 53 | 0.88 ± 0.02 | 0.73 ± 0.02 | 0.81 ± 0.01 | 0.89 ± 0.01 | 0.62 ± 0.01 |
| 49 | 0.88 ± 0.02 | 0.73 ± 0.02 | 0.80 ± 0.01 | 0.88 ± 0.01 | 0.61 ± 0.01 |
| 45 | 0.88 ± 0.02 | 0.72 ± 0.02 | 0.80 ± 0.01 | 0.88 ± 0.01 | 0.60 ± 0.01 |
| 33 | 0.86 ± 0.02 | 0.71 ± 0.02 | 0.79 ± 0.01 | 0.87 ± 0.01 | 0.58 ± 0.01 |
| 21 | 0.82 ± 0.02 | 0.71 ± 0.02 | 0.77 ± 0.01 | 0.84 ± 0.01 | 0.53 ± 0.01 |
| 9 | 0.74 ± 0.01 | 0.69 ± 0.02 | 0.71 ± 0.01 | 0.79 ± 0.01 | 0.43 ± 0.01 |

For Y, the results of 10-fold cross-validation are shown in **Table S4**. The relatively high standard deviations observed for this dataset suggest that there is more variability in performance, which is not surprising given the smaller size of the Y dataset compared to the other datasets. From **Figure 3,** MCC for Y does not follow specific pattern. For these reasons, the Chlamy-EnPhosSite and Chlamy-MwPhosSite models were not applied to the Y phosphorylation dataset, and an independent test was not performed.

Table S4. 10-fold cross-validation results for different windows for Y sites prediction.

| **Window Size** | **SN** | **SP** | **ACC** | **AUC** | **MCC** |
| --- | --- | --- | --- | --- | --- |
| 61 | 0.86 ± 0.09 | 0.76 ± 0.13 | 0.81 ± 0.06 | 0.81 ± 0.08 | 0.63 ± 0.13 |
| 57 | 0.83 ± 0.06 | 0.84 ± 0.13 | 0.83 ± 0.06 | 0.85 ± 0.06 | 0.66 ± 0.12 |
| 53 | 0.82 ± 0.17 | 0.80 ± 0.12 | 0.82 ± 0.07 | 0.83 ± 0.06 | 0.64 ± 0.13 |
| 49 | 0.87 ± 0.08 | 0.77 ± 0.16 | 0.83 ± 0.07 | 0.84 ± 0.13 | 0.66 ± 0.14 |
| 45 | 0.84 ± 0.10 | 0.74 ± 0.19 | 0.79 ± 0.08 | 0.82 ± 0.08 | 0.60 ± 0.16 |
| 33 | 0.83 ± 0.04 | 0.77 ± 0.12 | 0.81 ± 0.06 | 0.83 ± 0.07 | 0.61 ± 0.13 |
| 21 | 0.80 ± 0.08 | 0.76 ± 0.16 | 0.79 ± 0.05 | 0.80 ± 0.08 | 0.57 ± 0.10 |
| 9 | 0.89 ± 0.09 | 0.70 ± 0.19 | 0.80 ± 0.05 | 0.83 ± 0.08 | 0.61 ± 0.10 |

**Performance of models trained on non-organism specific phosphorylation sites to predict phosphorylation sites in *C. reinhardtii:***

Performance assessment of models trained on non-organism specific sites (MusiteDeep dataset^1^) was done with an independent test on phosphorylation sites in *C. reinhardtii*. For this, we used cross-learning where models are trained on the non-organism specific phosphorylation dataset and tested on the *C. reinhardtii* dataset (combined phosphorylation site dataset of S and T). The results are shown in **Table S5**.

Table S5. Performance metrics of different models in cross-learning using an independent test dataset for S and T. LSTM and CNN are our models trained on MusiteDeep dataset (non-organism specific phosphorylation sites).

| **Models** | **Sensitivity** | **Specificity** | **Accuracy** | **AUC** | **MCC** |
| --- | --- | --- | --- | --- | --- |
| LSTM with embedding (Trained on MusiteDeep Dataset) | 0.75 | 0.74 | 0.74 | 0.83 | 0.49 |
| CNN with embedding (Trained on MusiteDeep Dataset) | 0.79 | 0.72 | 0.75 | 0.83 | 0.52 |

1 Wang, D. *et al.* MusiteDeep: a deep-learning framework for general and kinase-specific phosphorylation site prediction. *Bioinformatics* **33**, 3909-3916, doi:10.1093/bioinformatics/btx496 (2017).

2 Luo, F., Wang, M., Liu, Y., Zhao, X. M. & Li, A. DeepPhos: prediction of protein phosphorylation sites with deep learning. *Bioinformatics*, doi:10.1093/bioinformatics/bty1051 (2019).

3 Lin, S. *et al.* Rice_Phospho 1.0: a new rice-specific SVM predictor for protein phosphorylation sites. *Scientific Reports* **5**, 11940, doi:10.1038/srep11940 (2015).
